# Human microglia express anti-inflammatory ISG15 in response to *Neisseria meningitidis*

**DOI:** 10.1016/j.neulet.2026.138543

**Published:** 2026-02-11

**Authors:** Andrew M. Dunphy, Krishna Majithia, Quinton A. Krueger, M. Brittany Johnson, Ian Marriott

**Affiliations:** Department of Biological Sciences, University of North Carolina at Charlotte, Charlotte, NC 28262, USA

**Keywords:** Human, Microglia, Astrocytes, *Neisseria meningitidis*, ISG15, Anti-inflammatory

## Abstract

Glial cells respond to the presence of bacteria by producing inflammatory mediators but these responses can result in damage to the central nervous system (CNS). However, glia can also produce immunosuppressive mediators that can serve to mitigate such effects. Here, we demonstrate that human microglial cells and, to a lesser extent, primary human astrocytes, can express and secrete interferon stimulated gene 15 (ISG15) in response to a clinically relevant CNS pathogen, *Neisseria meningitidis*, and ligands for Toll-like receptor 4 (TLR4) that include lipopolysaccharide and lipooligosaccharide derived from *N. meningitidis*. Exogenous ISG15 failed to elicit human neutrophil-like cell migration and induce or augment their inflammatory responses. Similarly, recombinant ISG15 application did not elicit inflammatory cytokine or chemokine production by either human microglial cells or astrocytes, and did not augment their responses to TLR stimulation or *N. meningitidis* infection. Rather, ISG15 treatment limited *N. meningitidis*-induced NF-κB activation and associated inflammatory cytokine production by these cells, perhaps via a non-canonical TLR-mediated pathway. These observations may be indictive of a novel negative feedback loop whereby the recognition of bacterial motifs precipitates ISG15 expression by resident microglia that subsequently mitigates further neuroinflammatory responses.

## 2. Introduction:

Glial cells rapidly respond to the presence of bacteria with the production of an array of inflammatory mediators that include chemokines and cytokines. Such responses influence the integrity of the blood brain barrier, and serve to recruit leukocytes and activate them upon arrival at the site of infection [1,2]. However, such responses can be detrimental if they are too severe or sustained, and this is of particular concern within the confines of the central nervous system (CNS). To mitigate the inflammatory damage associated with bacterial infection, microglia and astrocytes have been shown to express IL-10 and other members of this cytokine family, which function to limit the inflammatory responses of these cells and recruited immune cells [3–6].

The ubiquitin-like protein, interferon stimulated gene 15 (ISG15), is recognized to mediate antiviral immunity [7,8] and can promote inflammation association with viral infection [9,10]. In addition, ISG15 has been shown to be produced by leukocytes in response to bacterial lipopolysaccharide (LPS) [11] or *Mycobacterium tuberculosis* [12] and it has been reported to serve as a murine neutrophil chemotactic factor [13]. Human microglia in cerebral brain organoids show increased expression of mRNA encoding ISG15 following HIV infection [14]. However, ISG15 has also been shown to exert anti-inflammatory effects [15,16] and endogenously produced ISG15 has recently been reported to prevent TLR4-mediated NF-κB activation and associated inflammatory cytokine production by human microglia-like cells [17].

In the present study, we show that a human microglial cell line can express and secrete ISG15 in response to LPS, and a clinical strain of *N. meningitidis* and lipooligosaccharides (LOS) isolated from this organism. We have found that exogenous recombinant ISG15 can limit *N. meningitidis*-induced NF-κB activation and inflammatory mediator production in such cells. These observations may be indictive of a novel negative feedback loop whereby the recognition of bacterial motifs precipitate ISG15 expression by resident microglia that subsequently mitigates further neuroinflammation.

## 3. Materials and methods

A detailed description of the materials and methods can be found in the Supplemental Materials.

### 3.1. Source and propagation of human primary glia and cell lines

Primary human cortical astrocytes (ScienCell Research Laboratories) were cultured in the media supplied by the vendor. The human microglia cell line, hμglia (a generous gift from Dr. Jonathan Karn of Case Western Reserve University), was characterized and maintained as described [18–20]. Human leukemia-60 cells (HL-60) were differentiated to a mature neutrophil-like phenotype as described [21].

### 3.2. Bacterial propagation and infection of human cells

*Neisseria meningitidis* strain MC58 was grown and cultured prior to in vitro challenge as described [1,3,22,23], and the number of colony forming units (CFU) were determined by spectrophotometry. Glia or neutrophil-like cells were infected with bacteria at multiplicities of infection (MOI) ranging from 1 to 75 bacteria to each human cell in antibiotic-free medium for 2 h at 37°C with 5% CO_2_. These doses are based on bacterial numbers reported in the cerebral spinal fluid of bacterial meningitis patients [24] and our prior studies demonstrating the differing sensitivities of microglia and astrocytes to *N. meningitidis* challenge [1,3,22,23]. After infection, media containing penicillin–streptomycin was added to kill extracellular bacteria. We have confirmed that infection of glia with *N. meningitidis* at the highest MOI employed (50 and 75) significantly reduced cell viability at 24 h post-infection as assessed by colorimetric 3-(4,5-dimethylthiazol-2-yl)-5-(3-carboxymethoxyphenyl)-2-(4-sulfophenyl)–2H-tetrazolium (MTS) assay (Supplemental Fig. 3A). In some experiments, the total cell associated bacterial burden was assessed immediately following the 2-hour infection period and 24 h post infection by colony count of whole cell lysates.

### 3.3. Ligand stimulation

Glial cells were challenged with LPS, *N. meningitidis* LOS variants (92/89, 169/89, and 51/90) (generous gifts from Dr. Gary Jarvis of the University of California San Francisco), bacterial flagellin, or polyinosinic polycytidylic acid (polyI:C), or exposed to recombinant IFN-β (rISG15) or CXCL8. In some experiments, glial cells were co-treated with rISG15 at a dose (100 ng/mL) used previously [25,26], and endotoxin levels for this lot were determined by the vendor to be nominal (<960 EU/mg). We have confirmed that co-treatment of untreated or bacterially challenged glia with rISG15 failed to significantly reduce cell viability at 24 h as assessed by MTS assay (Supplemental Fig. 3A).

### 3.4. Isolation of RNA and semi-quantitative PCR

Total cellular RNA was isolated from cells and reverse transcribed for semi-quantitative PCR to determine expression of mRNA encoding ISG15 and the housekeeping gene human glyceraldehyde 3-phosphate dehydrogenase (GAPDH) as described [27] using verified primer pairs. ISG15 mRNA expression is reported as relative levels normalized to GADPH expression.

### 3.5. Immunoblot analyses

Cell lysates were evaluated by immunoblot analyses [28] for the presence of ISG15 or the housekeeping gene products, β-actin and tubulin, to assess total protein loading. Immunoblots shown are representative of at least three separate experiments and imaged for densitometric analysis.

### 3.6. Enzyme-linked immunosorbent assays (ELISAs)

Human ISG15, IL-6, IFN-β, CXCL1, and CXCL8 production was assessed using commercially available ELISA kits or antibody pairs.

### 3.7. Transwell^™^ cell migration assays

Neutrophil-like cells (1 × 10^6^) were added to the top of Transwell^™^ inserts of 24-well (pore size: 8 μm) plates and cell migration to the bottom well assessed at 3 h using an automated cell counter.

### 3.8. Nuclear translocation

Microglial cells were fractionated using a commercial kit (Cell Signaling) according to manufacturer’s guidelines, cleared of cellular debris by centrifugation, and supernatants containing the nuclear fraction were subjected to immunoblot analysis for NF-κB.

### 3.9. Transfection

Microglia were transfected with 5 nM control siRNA or siRNA targeted against ISG15 (Dharmacon) using RNAimax according to the manufacturer’s guidelines (ThermoFisher Scientific).

### 3.10. Statistical analysis

Data is expressed as the mean ± standard error of the mean (SEM). Commercially available software was used to conduct statistical analyses including Wilcoxon matched-pairs signed rank test and Dunn's post-hoc test with Bonferroni's correction for multiple comparisons, where a p-value of less than 0.0125 was considered statistically significant, or one- or two-way analysis of variance (ANOVA) with Šidák’s multiple comparisons test, where a p-value of less than 0.05 was considered statistically significant, as appropriate.

## 4. Results

### 4.1. Human microglial cells express ISG15 in response to N. meningitidis and LOS isolates

**Fig. 1.**
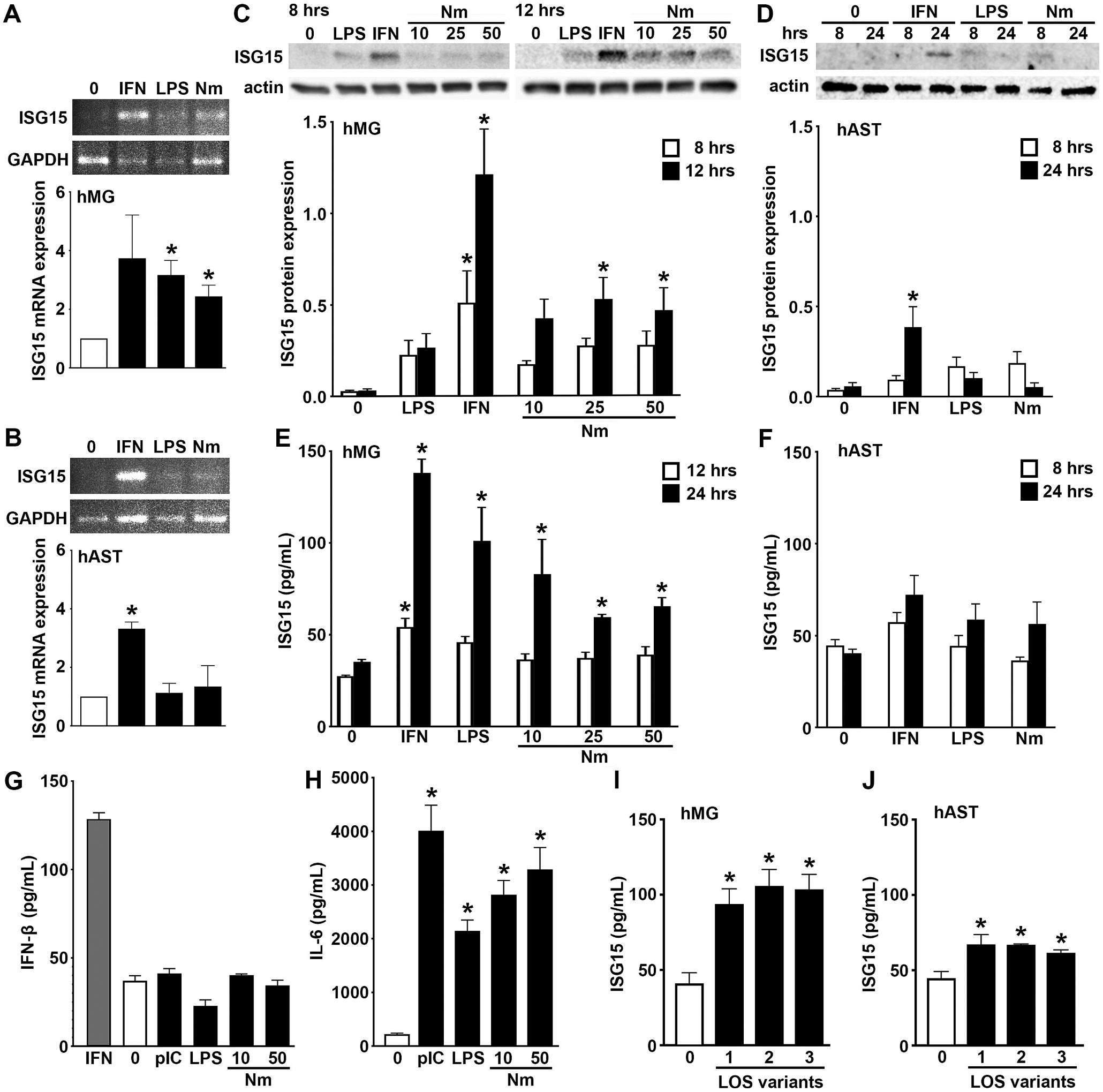
Human microglial cells express ISG15 in response to bacterial LPS, a clinical strain of *N. meningitidis*, and LOS isolates from this CNS pathogen. Microglial cells (hMG) and astrocytes (hAst) (5 × 10^4^ cell per well), were untreated (0), infected with *N. meningitidis* (Nm) at the indicated MOI, or exposed to stimuli including LPS, LOS, polyI:C, (pIC), or rIFN- β β (IFN) at the indicated concentrations. Panels A and B: Glia were untreated, infected with *N. meningitidis* (MOI of 1:50), or treated with LPS (10 ng/mL) or IFNβ (0.25 ng/mL (A) or 0.1 ng/mL (B)) for 4 h prior to RT-PCR for ISG15 or GAPDH. Blots shown are representatives of at least three independent experiments. The relative ISG15 expression was determined by densitometric analysis and normalized to untreated cells. Data is expressed as the mean +/− SEM of at least 3 independent experiments and an asterisk indicates a statistically significant difference from untreated cells by paired Student’s *t*-test (p < 0.05). Panels C-F: Microglia (C and E) and astrocytes (D and F) were untreated, infected with *N. meningitidis* (MOI of 10, 25 and 50 (C and E) or 50 only (D and F)), or treated with LPS (10 ng/mL) or IFN-β (0.25 ng/mL (C and E), 0.1 ng/mL (D and F)) for 8, 12, or 24 h prior to whole cell lysate and culture medium collection and analysis for the protein expression of ISG15 and β-actin (C and D) and ISG15 release (E and F), respectively. Immunoblots shown are representatives of at least three independent experiments. The relative ISG15 expression was determined by densitometric analysis and normalized to untreated cells. Data is expressed as the mean +/− SEM of at least 3 independent experiments and an asterisk indicates a statistically significant difference from untreated cells by two-way ANOVA (p < 0.05). Panels G and H: Microglia were untreated, infected with *N. meningitidis* (MOI of 10 and 50), or treated with LPS (10 ng/mL), polyI:C (500 ng/mL), or rIFN-β (0.25 ng/mL) for 24 h prior to analysis for IFN-β and IL-6 release. Data is expressed as the mean +/− SEM of at least 3 independent experiments and an asterisk indicates a statistically significant difference from untreated cells by one-way ANOVA (p < 0.05). Panels I and J: Microglia (I) and astrocytes (J) were untreated or treated with LOS variants 92/89, 169/89, and 51/90 (1, 2, and 3, respectively; 10 ng/mL) for 24 h prior to analysis for ISG15 release. Data is expressed as the mean +/− SEM of at least 3 independent experiments and an asterisk indicates a statistically significant difference from untreated cells by one-way ANOVA (p < 0.05).

To determine if human glial cells express ISG15 in response to clinically relevant bacteria or their products, the human microglial cell line, hμglia, and primary human astrocytes were challenged with LPS and *N. meningitidis*, and the positive control stimulus IFN-β. As shown in Fig. 1A and B, N*. meningitidis* and LPS elicited a rapid increase in ISG15 mRNA expression by human microglial cells, but not astrocytes. We then confirmed that both LPS and *N. meningitidis* elicit time-dependent increases in cellular ISG15 protein levels and/or ISG15 release in microglial cells as determined by immunoblot analysis and ELISA, respectively (Fig. 1C and E). Consistent with our assessments of ISG15 mRNA expression, neither LPS nor *N. meningitidis* challenge resulted in significant ISG15 production or release by human astrocytes (Fig. 1D and F). We have confirmed that ISG15 production by microglial cells does not occur secondary to the production of IFN-β with the demonstration that these cells failed to produce detectable levels of this cytokine with either LPS or *N. meningitidis* at these low MOI, or with the TLR3 ligand polyI:C, at 8, 12, or 24 h (Fig. 1G and data not shown), despite the ability of all three stimuli to evoke an IL-6 response (Fig. 1H). Furthermore, we have determined that three *N. meningitidis* LOS variants can elicit significant ISG15 release by human microglial cells (Fig. 1I) and, to a lesser extent, astrocytes (Fig. 1J).

### 4.2. ISG15 can attenuate NF-κB-driven inflammatory glial responses to N. meningitidis

**Fig. 2.**
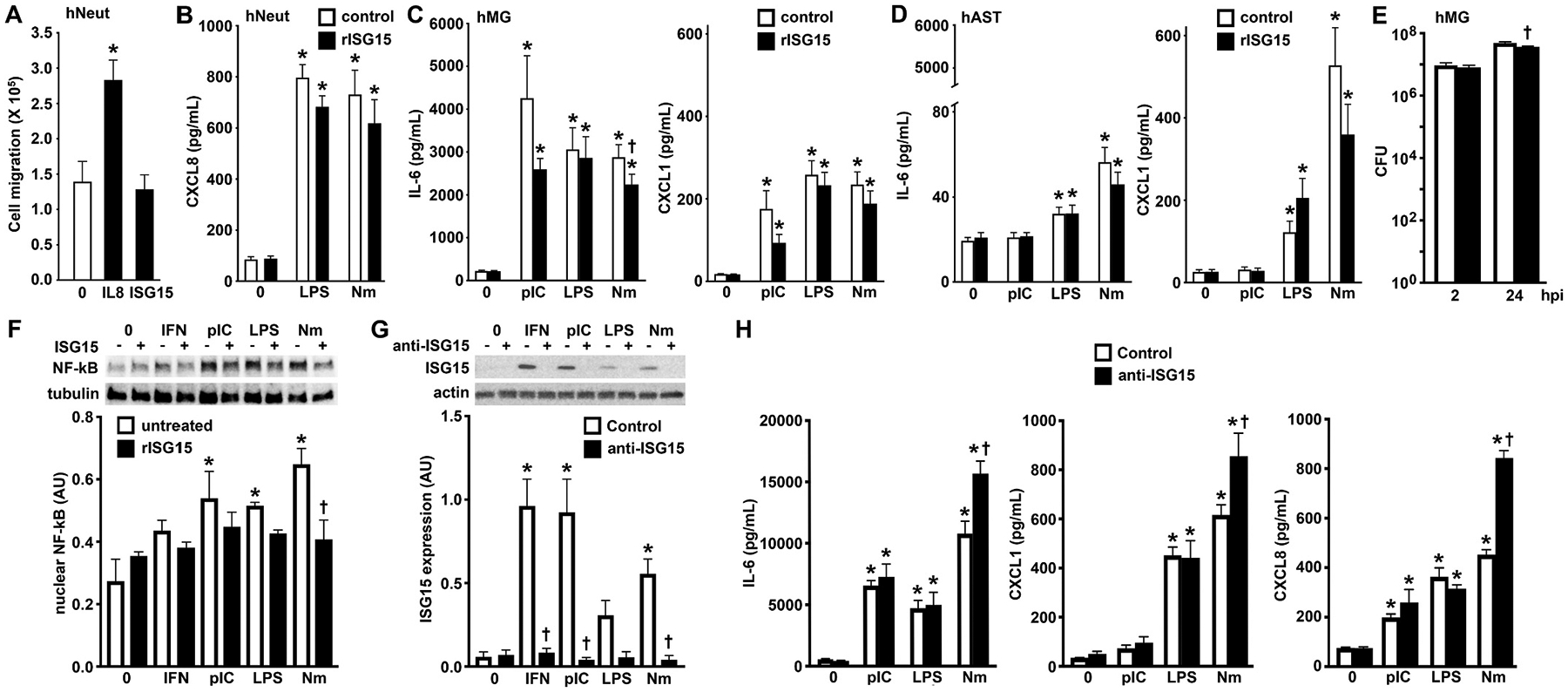
ISG15 can attenuate inflammatory glial responses to *N. meningitidis* and this effect is associated with a decrease in NF-κB activation. Panel A: rISG15 (1 ug/mL) fails to elicit neutrophil cell migration in Transwell^™^ apparatus in contrast to CXCL8 (IL8; 2 ng/mL). Panel B: Neutrophil-like cells (5 × 10^5^ cell per well), were untreated (0), infected with *N. meningitidis* (MOI of 10), or treated with LPS (10 ng/mL), in the absence or presence of rISG15 (100 ng/mL) for 24 h prior to analysis for CXCL8 release. Data is expressed as the mean +/− SEM of at least 3 independent experiments and an asterisk indicates a statistically significant difference from untreated cells by one-way ANOVA (A; p < 0.05) or Wilcoxon signed-rank test (B; p < 0.0125). Panels C-E: Microglia (C and E) and astrocytes (D) were untreated, infected with *N. meningitidis* (MOI of 10 and 50, respectively) or treated with polyI:C (0.5 μg/mL), or LPS (10 ng/mL), in the absence or presence of rISG15 (100 ng/mL) for 24 h prior to analysis for the release of IL-6 and CXCL1 (C and D), and 2 and 24 h prior to cell associated CFU enumeration (E). Data is expressed as the mean +/− SEM (n = 2–3), asterisks and dagger indicate a statistically significant difference from untreated cells and an effect of rISG15 on the response elicited by that stimulus, respectively, by Wilcoxon signed-rank test (p < 0.0125). Panel F: Microglia were untreated, infected with *N. meningitidis* (MOI of 50) or treated with IFN-β (IFN: 0.25 ng/mL), polyI:C (0.5 μg/mL), or LPS (5 ng/mL), in the absence or presence of rISG15 (100 ng/mL) for 1 h prior to nuclear extract preparation and immunoblot analysis for the presence of the NF-κB p65 subunit. Immunoblots shown are representatives of at least three independent experiments. The relative NF-κB p65 expression was determined by densitometric analysis and normalized to untreated cells. Data is expressed as the mean +/− SEM (n = 3). Asterisks and daggers indicate a statistically significant difference from untreated cells and similarly treated cells in the absence of rISG15, respectively, by one-way ANOVA (p < 0.05). Panels G and H: Microglia were transfected with control siRNA (control) or siRNA directed against ISG15 prior to being untreated or infected with *N. meningitidis* (MOI of 50) or treated with IFN-β (IFN: 0.25 ng/mL), polyI:C (0.5 μg/mL), or LPS (5 ng/mL) for 24 h prior to analysis for ISG15 and β-actin protein expression (G) or IL-6, CXCL1, and CXCL8 release (H). Immunoblots shown are representatives of at least three independent experiments. The relative ISG15 expression was determined by densitometric analysis and normalized to untreated cells. Data is expressed as the mean +/− SEM (n = 3). Asterisks and daggers indicate a statistically significant difference from untreated cells and similarly treated cells in the absence of ISG15 siRNA by two-way ANOVA (p < 0.05).

ISG15 is reported to have diverse activities that include serving as a neutrophil chemoattractant [13], a proinflammatory stimulus [9], and an immunosuppressive factor [15–17]. To begin to determine the functional significance of human glial production of ISG15 in response to bacterial infection, we have assessed the ability of ISG15 to induce migration of dHL-60 human neutrophil-like cells in a Transwell^™^ apparatus. As shown in Fig. 2A, recombinant ISG15 failed to elicit neutrophil migration in contrast to the chemoattractant CXCL8. Similarly, exogenous ISG15 failed to induce inflammatory chemokine production by these cells, or augment their inflammatory responses to LPS or *N. meningitidis* (Fig. 2B). Indeed, recombinant ISG15 tended to modestly attenuate LPS and *N. meningitidis* mediated CXCL8 production by these neutrophil-like cells (Fig. 2B).

Consistent with these findings, exogenous ISG15 failed to induce the production of IL-6 or CXCL1 by human microglial cells or astrocytes but significantly attenuated *N. meningitidis* stimulated IL-6 production by microglia and tended to decrease inflammatory mediator release by astrocytes (Fig. 2C and D). In addition, we have found that ISG15 can reduce *N. meningitidis*-induced CXCL8 production by microglial cells, albeit at a higher bacterial MOI (50:1; 990 +/− 68 pg/mL in untreated infected cells versus 825 +/− 60 pg/mL in ISG15 treated infected cells, n = 9, p < 0.05 with paired Student’s *t* test). The ability of ISG15 to attenuate inflammatory mediator production by microglia was not associated with an effect on total bacterial burden immediately following infection (Fig. 2E), but rISG15 treatment did result in a statistically significant, albeit modest, reduction in total bacterial burden of microglia at 24 h (Fig. 2E). Furthermore, the rISG15 mediated inhibition of *N. meningitidis*-induced cytokine release by microglia was unaffected by the STAT1 inhibitor fludarabine (10 ng/mL; Selleck Chem), arguing against a role for type I IFNs in these effects (Supplemental Fig. 3B). Interestingly, ISG15 also appeared to attenuate TLR ligand-mediated glial cell responses, reducing responses caused by the TLR3 ligand polyI:C in microglial cells, although this effect showed greater variability (Fig. 2C).

Consistent with a recent study in human glial cell lines [17], the ability of ISG15 to attenuate bacterially induced cytokine responses was found to correlate with a reduction in NF-κB activation as determined by diminished nuclear translocation of this master inflammatory regulator (Fig. 2F). Finally, we have assessed the impact of endogenously produced ISG15 on the inflammatory responses of human microglia using an siRNA approach. As shown in Fig. 2G, transfection of human microglia-like cells with siRNA directed against ISG15 abolished the increased expression of this molecule following stimulation with *N. meningitidis* or TLR ligands. Importantly, siRNA attenuation of bacterially-induced ISG15 levels markedly increased *N. meningitidis*-mediated production of IL-6, CXCL1, and CXCL8 by human microglia (Fig. 2H). Such a finding supports the notion that endogenous ISG15 production may serve to limit potentially detrimental NF-κB-driven inflammatory glial responses.

## 5. Discussion

ISG15 is known to be produced by, and mediate antiviral immunity in, many immune and non-immune cell types following viral infection [8]. However, ISG15 can also be induced in cells by bacterial challenge. For example, ISG15 expression has been associated with leukocytic responses to *Mycobacteria tuberculosis* and *Streptococcus pneumoniae* [4,29], and is expressed by fibroblasts in response to *Borrelia burgdorferi* [30]. With regard to glial cells, increased ISG15 mRNA expression has been shown in HIV infected microglia in human brain organoids [8] and a murine microglial cell line following rabies virus infection [31]. Interestingly, rabies virus has been reported to induce ISG15 protein expression in human iPSC-derived microglia but fails to do so in astrocytes [32]. In the present study, we have determined that a human microglia cell line can produce and secrete ISG15 in response to a clinical strain of *N. meningitidis*. Consistent with the negative findings in rabies virus infected astrocytes [32], the ability of human astrocytes to produce ISG15 in response to bacterial challenge was more equivocal, with low level expression of mRNA encoding this molecule seen following *N. meningitidis* challenge and only modest levels of protein expression and release. However, exposure of these cells to purified LOS isolated from *N. meningitidis* was capable of eliciting demonstrable ISG15 secretion, albeit in small amounts.

ISG15 expression is canonically induced via IFN signaling [8]. In this study, we show that infection with *N. meningitidis* induces ISG15 mRNA expression by microglia within four hours, which is inconsistent with an IFN-dependent secondary effect. More importantly, we have shown that the relatively low *N. meningitidis* MOI used in these studies fail to elicit demonstrable IFN-β production by these cells, despite being a potent stimulus for inflammatory cytokine release. Non-canonically, ISG15 has been suggested to be induced in an IFN-independent manner via the intracellular recognition of bacterial DNA [8,33] and *S. pneumoniae*-DNA has been reported to elicit ISG15 expression in murine monocyte-like cells [29]. However, ISG15 gene expression has also been shown to be upregulated by the TLR4 ligand LPS in human monocytes [8,11] and a TLR3 ligand in human stem cell-derived microglia [34]. Our studies provide support for such findings with the demonstration that the TLR4 ligands, LPS and LOS variants derived from *N. meningitidis*, and the TLR3 ligand, polyI:C, can induce ISG15 production by human microglial cells. However, further studies will be required to definitively establish the presence of such a non-canonical induction pathway in glial cells.

The significance of ISG15 expression by glial cells has been inadequately defined beyond its protective role in antiviral immunity. Exogenous ISG15 was reported to elevate the expression of iNOS and induce inflammatory cytokines production in murine microglia [35], and increased levels of ISG15 have been associated with a reduced infectious burden in *M. tuberculosis* infected murine microglia-like cells [36]. Furthermore, ISG15 has been suggested to serve as a neutrophil chemotactic factor [13]. However, our results do not support such effects as exogenous ISG15 neither elicited the migration of human neutrophil-like cells, nor induced inflammatory cytokine or chemokine production by these cells or microglia or astrocytes. Furthermore, we have found that the presence of extracellular ISG15 does not augment the release of these immune mediators following stimulation with TLR ligands or *N. meningitidis* in neutrophils or either glial cell type.

In contrast, some studies have reported that ISG15 can exert anti-inflammatory effects [15,16]. Our results support such a role with the demonstration that exogenous ISG15 can reduce inflammatory human microglial responses and modesty reduce intracellular bacterial burden at 24-hours following *N. meningitidis* infection, without affecting initial bacterial uptake. In addition, we have found that the presence of ISG15 tend to reduce cytokine and/or chemokine production by human glial cells triggered by ligands for TLR3. Interestingly, endogenous ISG15 has previously been shown to prevent TLR4-mediated NF-κB activation and associated IL-1β and IL-6 production by human microglia-like cells [17], and we have found that exogenous recombinant ISG15 can similarly inhibit *N. meningitidis*-induced NF-κB activation. Furthermore, we have shown that siRNA knockdown of endogenous ISG15 production by human microglial cells can significantly augment the release of IL-6, CXCL1, and CXCL8, inflammatory mediators whose production is largely driven by NF-κB, supporting an anti-inflammatory role during *N. meningitidis* infection.

Together, our data demonstrate that human microglia, more so than astrocytes, can express and secrete ISG15 in response to the clinically relevant CNS pathogen *N. meningitidis*, perhaps via a non-canonical TLR-mediated pathway. Endogenous and exogenous ISG15 exerts an anti-inflammatory effect on human microglial cells, limiting *N. meningitidis*-induced NF-κB activation and associated inflammatory cytokine production by these cells. Based on these observations, we suggest that induced ISG15 production by microglia serves to limit damaging neuroinflammation associated with this bacterium and potentially other neurotrophic Gram-negative bacteria. ISG15 could act together with other immunosuppressive mediators, such as IL-10 and related cytokines [5], in a negative feedback manner and we have recently discussed the potential role and implications for such a mechanism elsewhere [37]. However, future studies using primary human glial cells and featuring approaches such as co-culture will be required to definitively establish the role played by ISG15 in glia-neutrophil crosstalk.

## Supplementary Material

Raw blots

Supplemental figure 1

Supplemental methods

## Data Availability

Data will be made available on request.

## Significance statement:

Human glia secrete ISG15 in response to *Neisseria meningitidis* that may serve to limit damaging neuroinflammation.

## Appendix A. Supplementary data

Supplementary data to this article can be found online at https://doi.org/10.1016/j.neulet.2026.138543.
