# Supplementary material for: Human microglia express anti-inflammatory ISG15 in response to Neisseria meningitidis
Source: Neurosci Lett. Author manuscript; Available in PMC 2026 Jul 9. (PMC13347968; doi:10.1016/j.neulet.2026.138543)
Supplement: Raw blots [file NIHMS2192923-supplement-Raw_blots.pdf]

ANNOTATED IMMUNOBLOTS USED IN FIGURES 1 AND 2

FIGURES 1A-D: Immortalized human microglial cells (hMG) and primary human (hAst) (5 X 10^4 cell per well), were untreated (0), infected with *N. meningitidis* (Nm) at the indicated MOI of bacteria to each mammalian cell, or exposed to stimuli including LPS, LOS, polyI:C, (pIC), or recombinant IFN-b (IFN) at the indicated concentrations. Panels A and B: Glial cells were untreated, infected with *N. meningitidis* (MOI of 1:50), or treated with LPS (10 ng/mL) or IFN-b (0.25 ng/mL (A) or 0.1 ng/mL (B)) for 4 hours prior to semi-quantitative RT-PCR for ISG15 or the housekeeping gene product GAPDH. Panels C and D: Microglia (C) and astrocytes (D) were untreated, infected with *N. meningitidis* (MOI of 10, 25 and 50 (C) or 50 only (D)), or treated with LPS (10 ng/mL) or IFN-b (0.25 ng/mL (C), 0.1 ng/mL (D)) for 8, 12, or 24 hours prior to whole cell lysate collection and analysis for the expression of ISG15 and β-actin by immunoblot analysis (C and D).

0 IFN LPS Nm

1000 bp

500 bp

100 bp

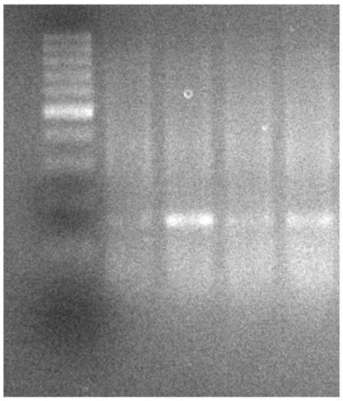

FIGURE 1A mRNA encoding ISG15 in microglia

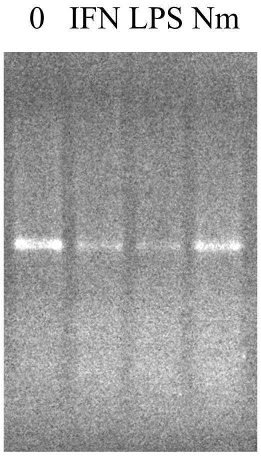

FIGURE 1A GAPDH mRNA expression in microglia

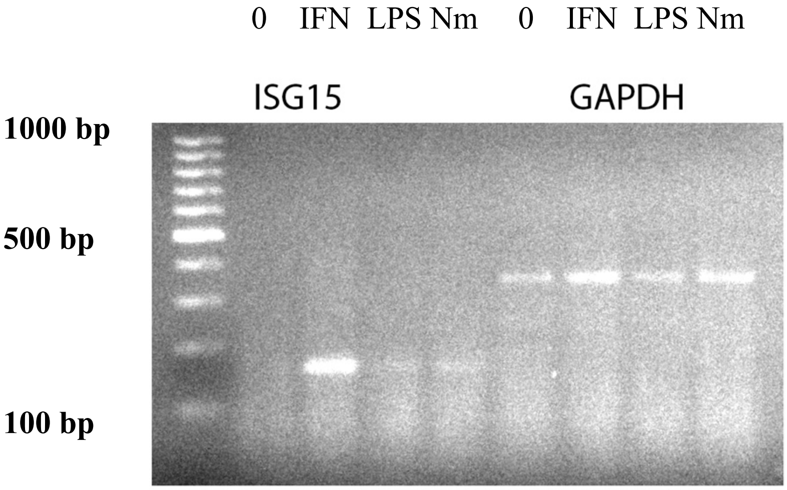

FIGURE 1B ISG15 and GAPDH mRNA expression in astrocytes

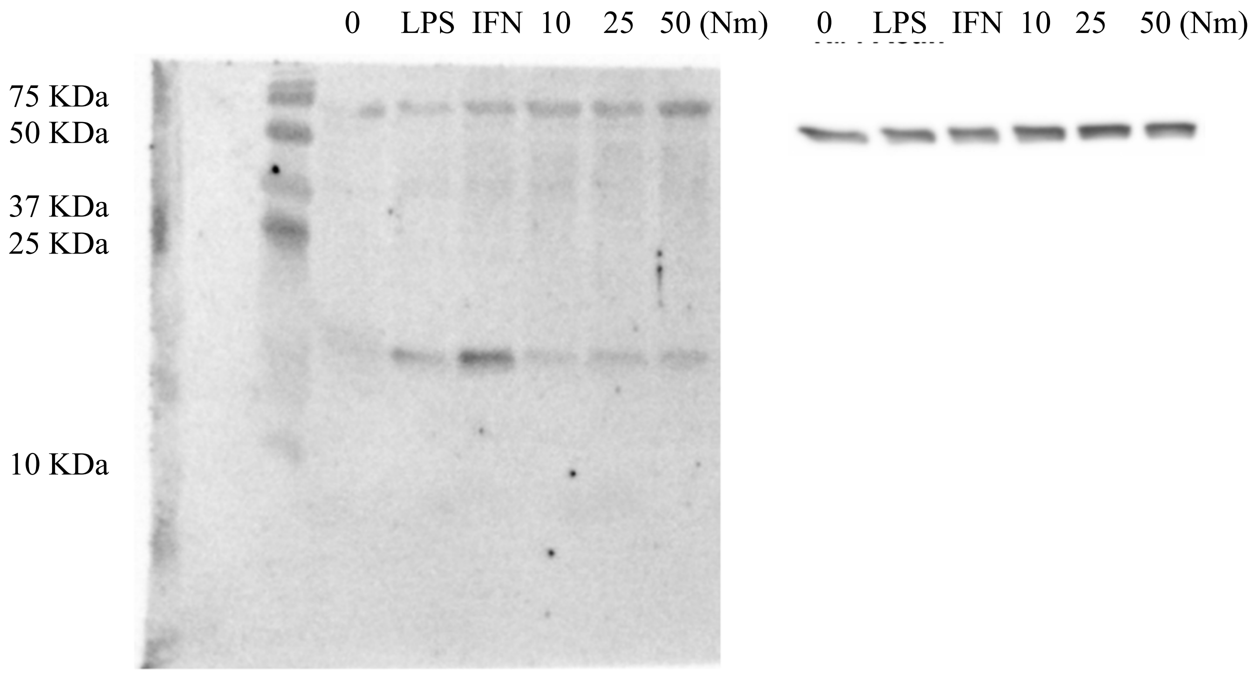

FIGURE 1C ISG15 and beta-actin protein expression in microglia at 8 hrs

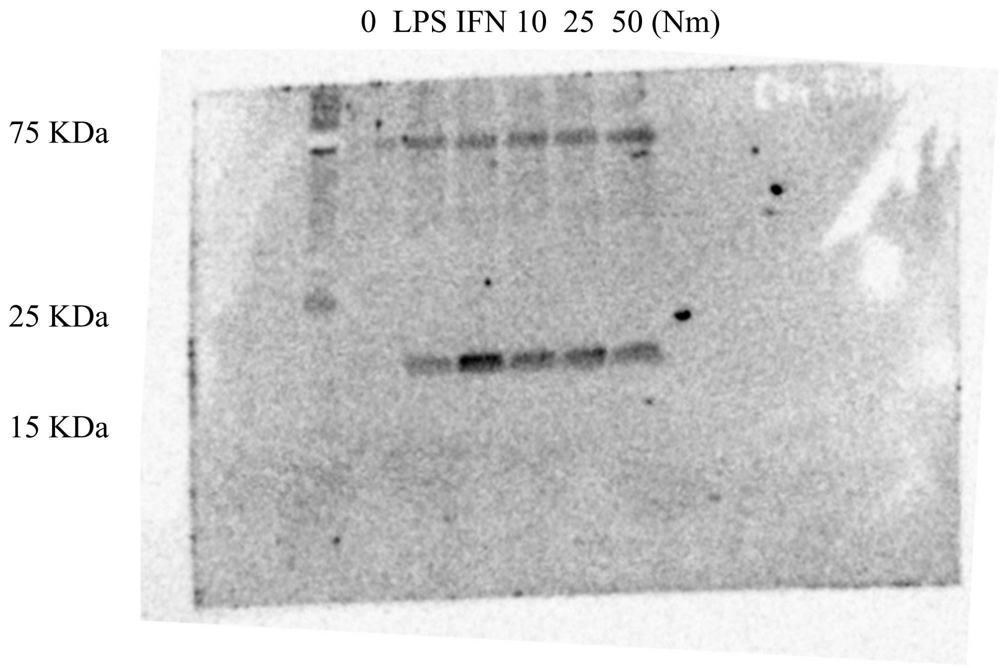

FIGURE 1C ISG15 protein expression in microglia at 12 hrs.

0 LPS IFN 10 25 50 (Nm)

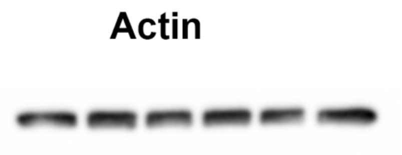

FIGURE 1C beta-actin protein expression in microglia at 12 hrs.

8 24 8 24 8 24 8 24 hrs
0 0 IFN IFN LPS LPS Nm Nm

50 KDa
20 KDa
15 KDa

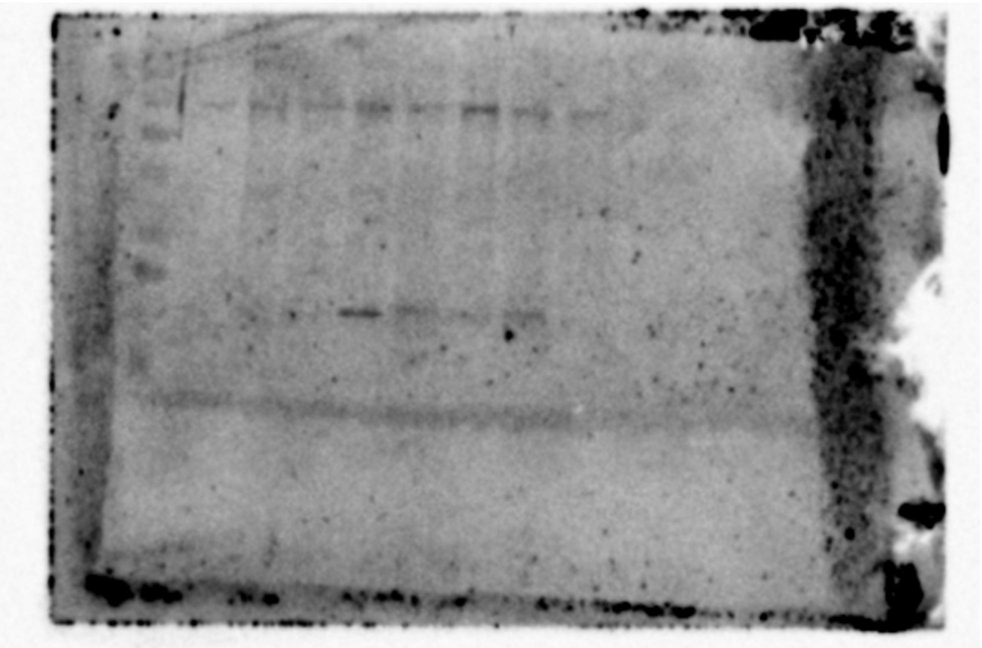

FIGURE 1D ISG15 protein expression in astrocytes

8 24 8 24 8 24 8 24 hrs
0 0 IFN IFN LPS LPS Nm Nm

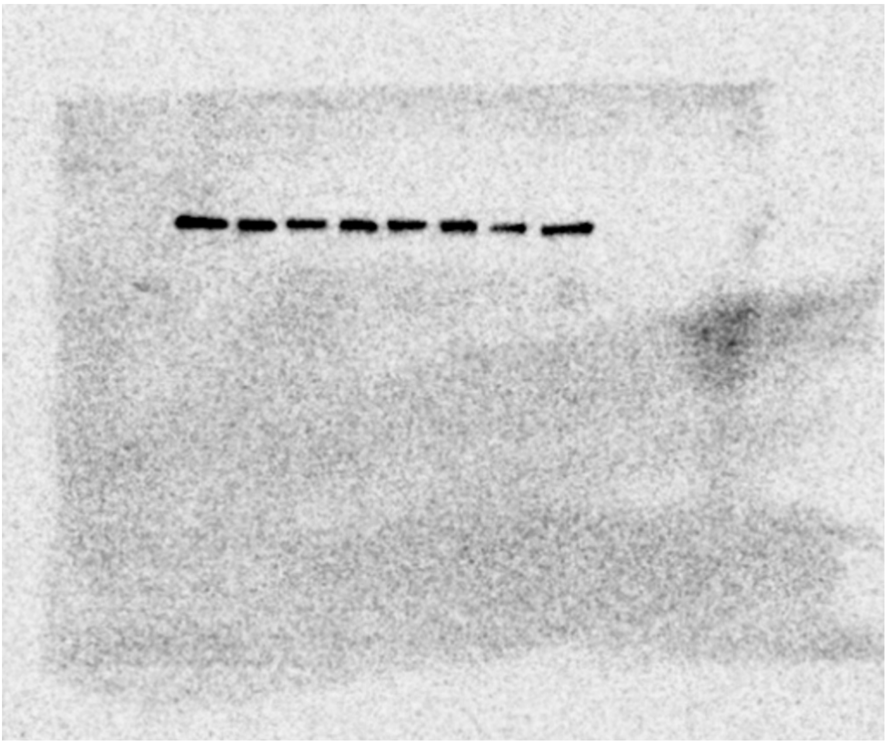

FIGURE 1D beta-actin protein expression in astrocytes

FIGURES 2F and G: Panel F: Microglia were untreated, infected with *N. meningitidis* (MOI of 50) or treated with IFN-beta (IFN: 0.25 ng/mL), polyI:C (0.5 μg/mL), or LPS (5 ng/mL), in the absence or presence of recombinant ISG15 (100 ng/mL) for 1 hour prior to nuclear extract preparation and immunoblot analysis for the presence of the p65 subunit of NF-kB. Panel G: Microglia were untransfected or transfected with siRNA directed against ISG15 prior to being untreated or infected with *N. meningitidis* (MOI of 50) or treated with IFN-beta (IFN: 0.25 ng/mL), polyI:C (0.5 μg/mL), or LPS (5 ng/mL) for 24 hours prior to whole cell protein isolation (G) and immunoblot analysis for the presence of ISG15 and beta-actin.

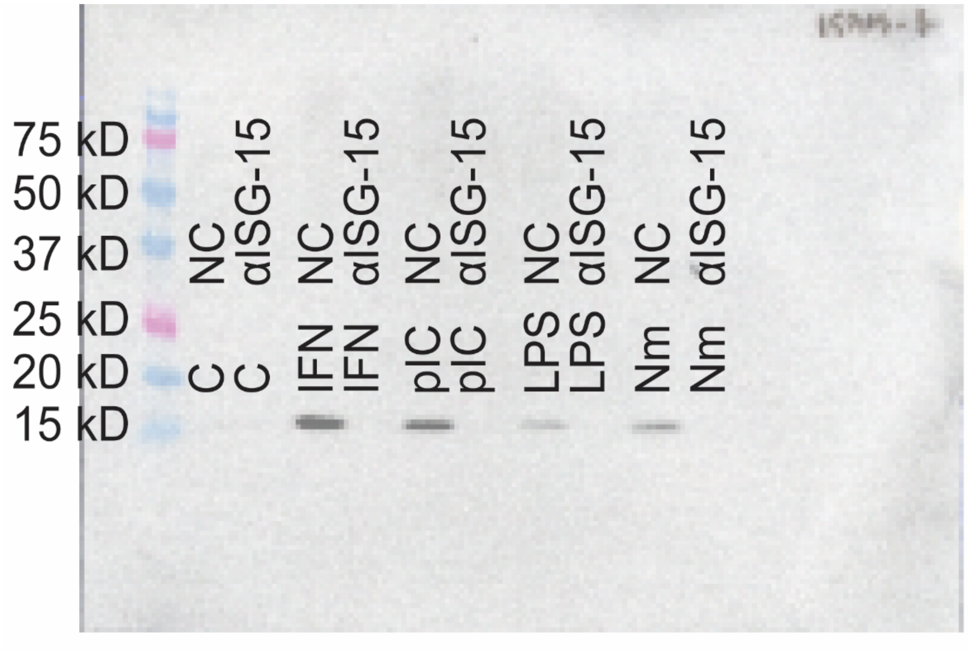

FIGURE 2H ISG15 protein expression in microglia

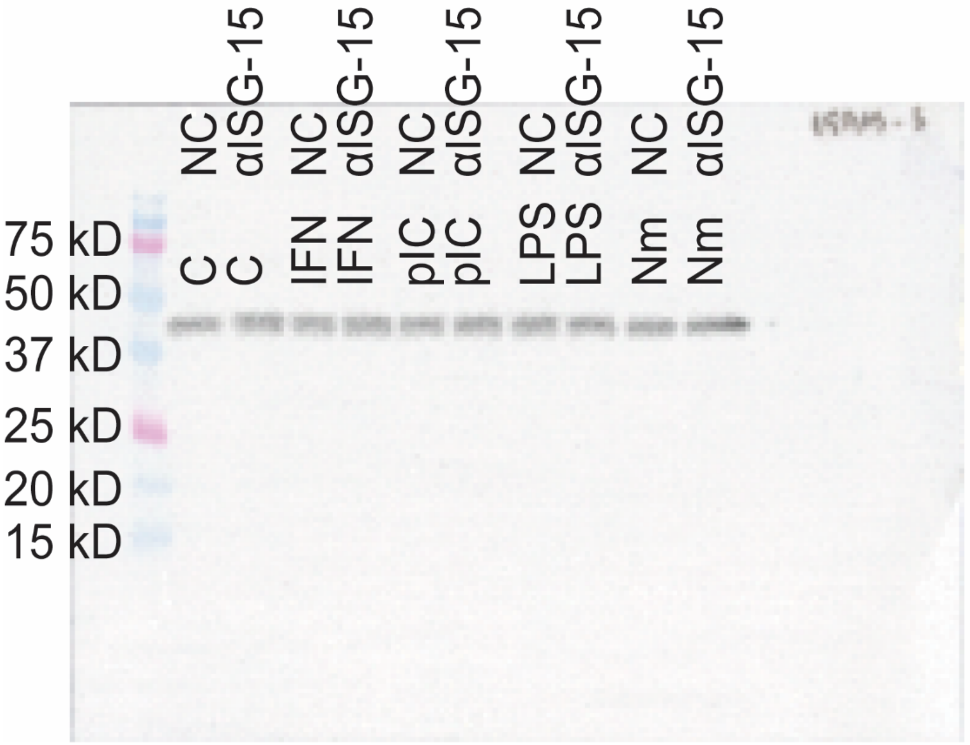

FIGURE 2H beta-actin protein expression in microglia

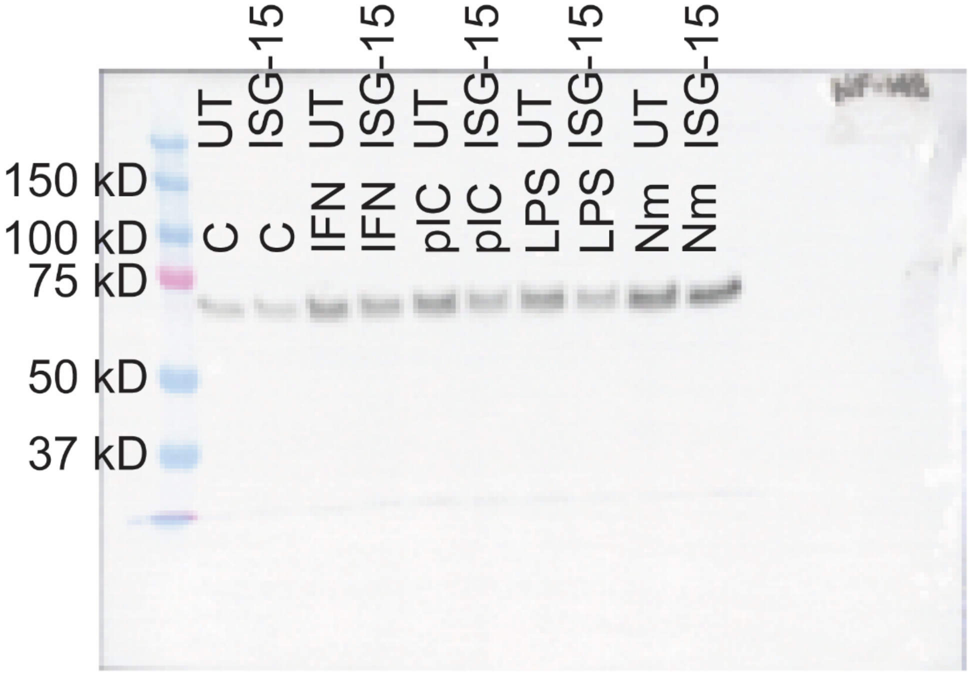

FIGURE 2G Nuclear NF-kB p65 expression in microglia

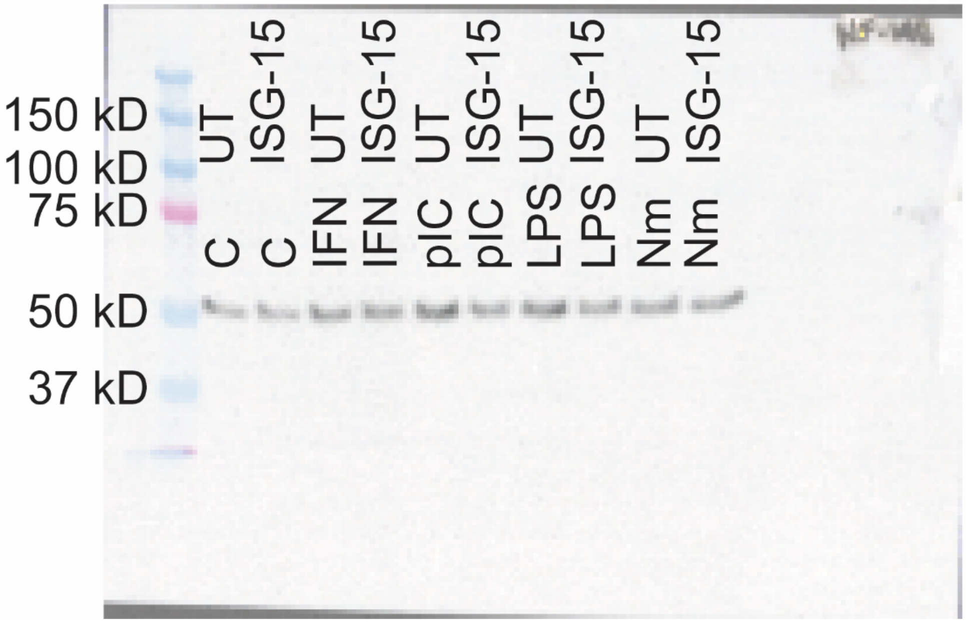

FIGURE 2G Nuclear tubulin expression in microglia
