# Supplementary figures and images for: Human microglia express anti-inflammatory ISG15 in response to Neisseria meningitidis
Source: Neurosci Lett. Author manuscript; Available in PMC 2026 Jul 9. (PMC13347968; doi:10.1016/j.neulet.2026.138543)

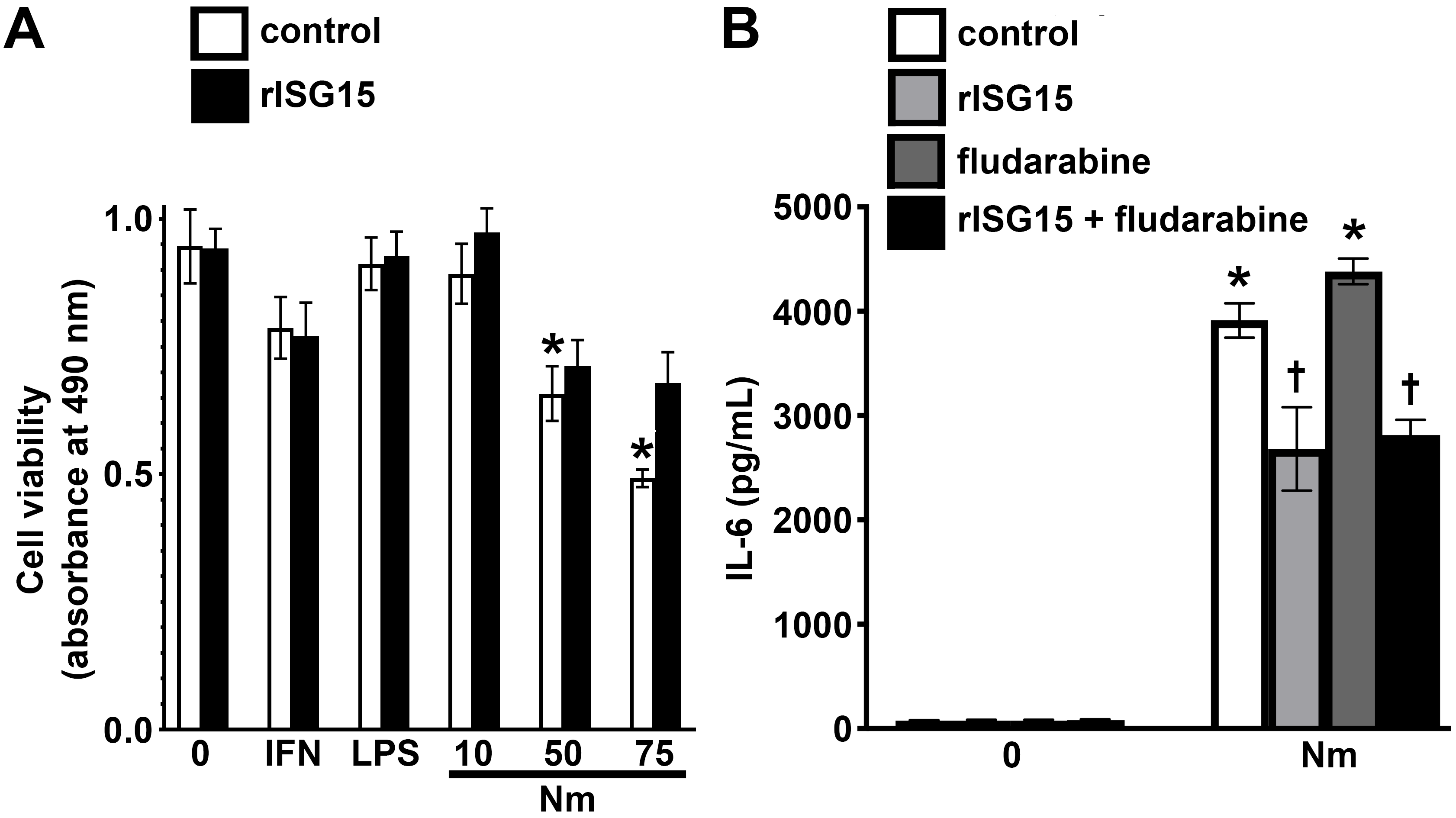

Supplement: Supplemental figure 1 [file NIHMS2192923-supplement-Supplemental_figure_1.tif]
