# Supplementary material for: Human microglia express anti-inflammatory ISG15 in response to Neisseria meningitidis
Source: Neurosci Lett. Author manuscript; Available in PMC 2026 Jul 9. (PMC13347968; doi:10.1016/j.neulet.2026.138543)
Supplement: Supplemental methods [file NIHMS2192923-supplement-Supplemental_methods.docx]

**DETAILED MATERIALS AND METHODS**

*Source and propagation of human primary glia and cell lines*

Primary human cortical astrocytes, characterized as glial fibrillary acidic protein (GFAP) positive by immunofluorescence and cryopreserved at passage one, were purchased from ScienCell Research Laboratories (Carlsbad, CA). These cells were cultured in the media supplied by the vendor and were used up to passage 10 in accordance with their recommendations. The human microglia cell line, hµglia, was a generous gift from Dr. Jonathan Karn (Case Western Reserve University). These cells were derived from primary human cells transformed with lentiviral vectors expressing SV40 T antigen and hTERT. These cells have been classified as microglia due to their microglia-like morphology, migratory and phagocytic activity, presence of the microglial surface markers, CD11B, TGFβR, and P_2_RY_12_, and a characteristic microglial RNA expression profile [1-3]. Cells were maintained in Dulbecco’s Modified Eagle’s Medium (DMEM) supplemented with 5% FBS and 100 U/mL penicillin-100 µg/mL streptomycin at 37°C 5% CO_2._

*Source and propagation of a human neutrophil cell line*

Human leukemia-60 cells (HL-60; ATCC) were differentiated to mature neutrophil-like cells in Iscove’s DMEM with 20% lot-tested fetal bovine serum (FBS), 1% 1X Glutamax (Invitrogen Cat# 35050061), 1% penicillin/streptomycin, and 1.25% DMSO for 5 days as we have previously described [4]. We have previously confirmed the mature neutrophil-like phenotype of these cells using flow cytometry to confirm increased expression of the mature neutrophil differentiation markers CD11b and CD35, but low levels of the immature neutrophil marker CD71 as we previously described [4].

*Bacterial propagation and infection of human cells*

*Neisseria meningitidis* strain MC58 (ATCC BAA-335) was grown on Columbia agar plates supplemented with 5% defibrinated sheep blood (BD, Franklin Lakes, NJ) and cultured in Columbia broth (BD Biosciences, San Jose, CA) on an orbital rocker at 37°C with 5% CO_2_ overnight prior to in vitro challenge. The number of colony forming units (CFU) were determined by spectrophotometry using a Thermo Scientific NanoDrop OneC microvolume spectrophotometer. Human glia or neutrophil-like cells were infected with bacteria at multiplicities of infection (MOI) ranging from 1 to 50 bacteria to each human cell in antibiotic-free medium for 2 h at 37ºC with 5% CO_2_. These doses are based on bacterial numbers previously reported in the cerebral spinal fluid of children with bacterial meningitis [5] and our prior studies demonstrating the differing relative sensitivities of microglia and astrocytes to *Neisseria meningitidis* challenge [6-9]. After 2 hours of infection, media containing penicillin-streptomycin (MilliporeSigma, St. Louis, MO) was added to kill extracellular bacteria. We have confirmed that infection of glia with *N. meningitidis* at the highest MOI employed (50 and 75 bacteria to each human cell) significantly reduce cell viability at 24 hours post infection as assessed by colorimetric 3-(4,5-dimethylthiazol-2-yl)-5-(3-carboxymethoxyphenyl)-2-(4-sulfophenyl)-2H-tetrazolium (MTS) assay (Supplemental Figure 1A). At the indicated time points following challenge, cell supernatants, whole cell protein lysates, and RNA, were isolated for ELISAs, immunoblot analysis, and RT-PCR, respectively. In some experiments, the total bacterial burden was assessed in glia immediately following the 2-hour infection period and 24 hours post infection by colony count of whole cell lysates to assess effects on the bacterial internalization process and survival.

*Ligand stimulation*

Glial cells were exposed to LPS isolated from *Escherichia coli* (Cell Signaling), LOS variants (92/89, 169/89, and 51/90) isolated from *N. meningitidis* (generous gifts from Dr. Gary Jarvis of the University of California San Francisco), bacterial flagellin isolated from *Salmonella typhimurium strain* LT2 (Abcam), or polyinosinic polycytidylic acid (polyI:C; Fisher Scientific and VWR), or exposed to recombinant IFN-β (R&D Systems or GenScript) or CXCL8 (R&D Systems). In some experiments, glial cells were co-treated with recombinant ISG15 (rISG15; 100 ng/mL; ThermoFisher Scientific, Cat# RP88081, Lot# ZI4468682). This dose of rISG15 was selected based upon prior studies in other cell types [10,11] and endotoxin levels for this lot were determined by the vendor to be nominal (<960 EU/mg). We have confirmed that co-treatment of untreated or bacterially challenged glia with rISG15 failed to significantly reduce cell viability at 24 hours post infection as assessed by MTS assay (Supplemental Figure 1A).

*Isolation of RNA and semi-quantitative PCR*

Total cellular RNA was isolated from microglia and astrocytes with TRIzol reagent (ThermoFisher) and reverse transcribed as previously described [12]. Semi-quantitative PCR was performed to determine expression or transcript length/composition of mRNA encoding human glyceraldehyde 3-phosphate dehydrogenase (GAPDH) and ISG15 as we have described [12]. PCR primers used to amplify cDNA derived from cellular mRNA were as follows: ISG15 forward (GTG GAC AAA TGC GAC GAA CC), ISG15 reverse (TCG AAG GTC AGC CAG AAC AG), GAPDH forward (CCA TCA CCA TCT TCC AGG AGT GAG), GAPDH reverse (CAC AGT CTT CTG GGT GGC AGT GAT). These primers were designed using commercial software (Integrated DNA Technologies, Coralville, IA) based on their location in different exons of the genomic sequences and their lack of significant homology to sequences present in GenBank (National Center for Biotechnology Information, Bethesda, MD). The identity of each PCR amplified fragment was verified by size comparison with DNA standards (Promega, Madison, WI). All RNA expression levels are reported as relative levels normalized to the expression of the housekeeping gene GADPH determined in parallel PCR reactions.

*Immunoblot analyses*

Cell lysates were evaluated for the presence of ISG15 by immunoblot analyses [13]. Blots were incubated with a rabbit polyclonal antibody against human ISG15 (Cell Signaling; 2743S) overnight at 4°C. Blots were then washed and incubated in the presence of a horseradish peroxidase (HRP)-conjugated secondary anti-rabbit antibody (Cell Signaling, 7074S). Bound antibody was detected with West Pico PLUS ECL kit (Thermo Scientific; 34580). Immunoblots were re-probed with a rabbit monoclonal antibody against β-actin (Cell Signaling; 4967S) or tubulin (Cell Signaling; 2146S) to assess total protein loading. Immunoblots shown are representative of at least three separate experiments and imaged using an Azure 300 imager with AzureSpotPro software for densitometric analysis.

*Enzyme-linked immunosorbent assays (ELISAs)*

To quantify human ISG15, IL-6, IFN-β, CXCL1 and CXCL8 production, specific capture ELISAs were performed. Commercially available ELISA kits were employed to measure ISG15 (Abcam; ab303764), IFN-β, CXCL1 and CXCL8 (R&D Systems; DY81405, DY275-05, and DY208-15, respectively) production according to the manufacturer’s directions. A rat anti-human IL-6 capture antibody (BD Pharmingen; Clone MQ2-13A5) and a biotinylated rat anti-human IL-6 detection antibody (BD Pharmingen; Clone MQ2-39C3) were used in IL-6 ELISAs. Bound antibody was detected using streptavidin-HRP (R&D Systems) followed by the addition of tetramethylbenzidine substrate (VWR; TMBW-1000-01). H_2_SO_4_ was used to stop the reaction and absorbance was measured at 450 nm. A standard curve was generated using dilution of recombinant cytokines for ISG15 (Abcam), IL-6 (BD Pharmingen), IFN-β, CXCL1, and CXCL8 (R&D Systems). The concentration of the molecule of interest in cell supernatants was determined by extrapolation of absorbance to the standard curve.

*Transwell^TM^ cell migration assays*

HL-60 neutrophil-like cells (1 X 10^6^) were added to the top of Transwell^TM^ (ThinCerts; Greiner Bio-One; Millicell Cell Culture Inserts, Millipore) inserts of 24-well (pore size: 8 µm) plates. Migration of these cells, in response to media alone, recombinant CXCL8 (R&D Systems) or ISG15 (Invitrogen) was assessed by counting the number of cells in the bottom well at 3 hours using an automated cell counter (Countess 3 FL; ThermoFisher).

*Nuclear translocation*

Microglial cells were fractionated using a cell fractionation kit (Cell Signaling) according to manufacturer’s guidelines. Briefly, following isolation of cytoplasmic and membrane fractions, nuclear fractions were isolated using the nuclear isolation buffer (CyNIB) for 5 min at 4°C, and lysed by sonication. Samples were cleared of cellular debris by centrifugation, and supernatants containing the nuclear fraction were subjected to immunoblot analysis using a specific rabbit monoclonal antibody for NF-κB (Cell Signaling; 8242S).

*Transfection*

Microglia were transfected with 5 nM control siRNA (Dharmacon; D-001810-10-05) or siRNA targeted against ISG15 (Dharmacon; L-004235-03-0005) for 24 hours. siRNA was transfected according to the manufacture’s guidelines using RNAimax (ThermoFisher Scientific).

*Statistical analysis*

Data is expressed as the mean ± standard error of the mean (SEM). Commercially available software (GraphPad Prism, La Jolla, CA) was used to conduct statistical analyses including Wilcoxon matched-pairs signed rank test and Dunn's post-hoc test with Bonferroni's correction for multiple comparisons, where a p-value of less than 0.0125 was considered statistically significant, or one- or two-way analysis of variance (ANOVA) with Šidák’s multiple comparisons test, where a p-value of less than 0.05 was considered statistically significant, as appropriate.

*References*

1. Garcia-Mesa Y, Jay TR, Checkley MA, Luttge B, Dobrowolski C, Valadkhan S, Landreth GE, Karn J, Alvarez-Carbonell D. Immortalization of primary microglia: a new platform to study HIV regulation in the central nervous system. J Neurovirol. 2017 Feb;23(1):47-66. doi: 10.1007/s13365-016-0499-3. Epub 2016 Nov 21. PMID: 27873219; PMCID: PMC5329090.

2. Burmeister AR, Johnson MB, Chauhan VS, Moerdyk-Schauwecker MJ, Young AD, Cooley ID, Martinez AN, Ramesh G, Philipp MT, Marriott I. Human microglia and astrocytes constitutively express the neurokinin-1 receptor and functionally respond to substance P. J Neuroinflammation. 2017 Dec 13;14(1):245. doi: 10.1186/s12974-017-1012-5. PMID: 29237453; PMCID: PMC5729418.

3. Jeffries AM, Marriott I. Human microglia and astrocytes express cGAS-STING viral sensing components. Neurosci Lett. 2017 Sep 29;658:53-56. doi: 10.1016/j.neulet.2017.08.039. Epub 2017 Aug 19. PMID: 28830822; PMCID: PMC5645252.

4. Sipprell SE, Johnson MB, Leach W, Suptela SR, Marriott I. Staphylococcus aureus Infection Induces the Production of the Neutrophil Chemoattractants CXCL1, CXCL2, CXCL3, CXCL5, CCL3, and CCL7 by Murine Osteoblasts. Infect Immun. 2023 Apr 18;91(4):e0001423. doi: 10.1128/iai.00014-23. Epub 2023 Mar 7. PMID: 36880752; PMCID: PMC10112169.

5. Bingen E, Lambert-Zechovsky N, Mariani-Kurkdjian P, Doit C, Aujard Y, Fournerie F, Mathieu H. Bacterial counts in cerebrospinal fluid of children with meningitis. Eur J Clin Microbiol Infect Dis. 1990 Apr;9(4):278-81. doi: 10.1007/BF01968060. PMID: 2112465.

6. Chauhan VS, Sterka DG Jr, Gray DL, Bost KL, Marriott I. Neurogenic exacerbation of microglial and astrocyte responses to Neisseria meningitidis and Borrelia burgdorferi. J Immunol. 2008 Jun 15;180(12):8241-9. doi: 10.4049/jimmunol.180.12.8241. PMID: 18523290; PMCID: PMC2507752.

7. Rasley A, Tranguch SL, Rati DM, Marriott I. Murine glia express the immunosuppressive cytokine, interleukin-10, following exposure to Borrelia burgdorferi or Neisseria meningitidis. Glia. 2006 Apr 15;53(6):583-92. doi: 10.1002/glia.20314. PMID: 16419089.

8. Johnson MB, Halman JR, Burmeister AR, Currin S, Khisamutdinov EF, Afonin KA, Marriott I. Retinoic acid inducible gene-I mediated detection of bacterial nucleic acids in human microglial cells. J Neuroinflammation. 2020 May 1;17(1):139. doi: 10.1186/s12974-020-01817-1. PMID: 32357908; PMCID: PMC7195775.

9. Burmeister AR, Johnson MB, Marriott I. Murine astrocytes are responsive to the pro-inflammatory effects of IL-20. Neurosci Lett. 2019 Aug 24;708:134334. doi: 10.1016/j.neulet.2019.134334. Epub 2019 Jun 22. PMID: 31238130; PMCID: PMC6693946.

10. Alcalá S, Sancho P, Martinelli P, Navarro D, Pedrero C, Martín-Hijano L, Valle S, Earl J, Rodríguez-Serrano M, Ruiz-Cañas L, Rojas K, Carrato A, García-Bermejo L, Fernández-Moreno MÁ, Hermann PC, Sainz B Jr. ISG15 and ISGylation is required for pancreatic cancer stem cell mitophagy and metabolic plasticity. Nat Commun. 2020 May 29;11(1):2682. doi: 10.1038/s41467-020-16395-2. PMID: 32472071; PMCID: PMC7260233.

11. Wang SH, Chen YL, Huang SH, Fu YK, Lin SF, Jiang SS, Liu SC, Hsiao JR, Chang JY, Chen YW. Tumor cell-derived ISG15 promotes fibroblast recruitment in oral squamous cell carcinoma via CD11a-dependent glycolytic reprogramming. Oncogenesis. 2025 Mar 11;14(1):6. doi: 10.1038/s41389-025-00549-2. PMID: 40069143; PMCID: PMC11897235.

12. Bowman CC, Rasley A, Tranguch SL, Marriott I. Cultured astrocytes express toll-like receptors for bacterial products. Glia. 2003 Sep;43(3):281-91. doi: 10.1002/glia.10256. PMID: 12898707.

13. Furr SR, Chauhan VS, Sterka D Jr, Grdzelishvili V, Marriott I. Characterization of retinoic acid-inducible gene-I expression in primary murine glia following exposure to vesicular stomatitis virus. J Neurovirol. 2008 Nov;14(6):503-13. doi: 10.1080/13550280802337217. PMID: 18991139; PMCID: PMC3833003.
